# Role of *p53* isoforms in the DNA damage response during *Drosophila* oogenesis

**DOI:** 10.1038/s41598-019-47913-y

**Published:** 2019-08-07

**Authors:** Ji-Hong Park, Tram Thi Ngoc Nguyen, Eun-Mi Lee, Veronica Castro-Aceituno, Ram Wagle, Kwang-Soon Lee, Juyoung Choi, Young-Han Song

**Affiliations:** 1 Department of Biomedical Gerontology, Hallym University, Chuncheon, Gangwon-do Republic of Korea; 2 Ilsong Institute of Life Science, Hallym University, Anyang, Gyeonggi-do Republic of Korea

**Keywords:** Apoptosis, Oogenesis

## Abstract

The tumor suppressor *p53* is involved in the DNA damage response and induces cell cycle arrest or apoptosis upon DNA damage. *Drosophila p53* encodes two isoforms, *p53A* and *p53B*, that induce apoptosis in somatic cells. To investigate the roles of *Drosophila p53* isoforms in female germline cells, the DNA damage response was analyzed in the adult ovary. Early oogenesis was sensitive to irradiation and *lok*-, *p53*-, and *hid*-dependent cell death occurred rapidly after both low- and high-dose irradiation. Both *p53* isoforms were responsible for this cell death. On the other hand, delayed cell death in mid-oogenesis was induced at a low level only after high-dose irradiation in a *p53*-independent manner. The daily egg production, which did not change after low-dose irradiation, was severely reduced after high-dose irradiation in *p53* mutant females due to the loss of germline stem cells. When the *p53A* or *p53B* isoform was expressed in the germline cells in the *p53* mutant females at levels that do not affect normal oogenesis, *p53A*, but not *p53B*, restored the fertility of the irradiated female. In summary, moderate expression of *p53A* is critical to maintain the function of germline stem cells during normal oogenesis as well as after high-dose irradiation.

## Introduction

Ionizing radiation (IR) activates the DNA damage response resulting in cell cycle arrest, DNA repair, apoptosis, and senescence^1^. *Chk2* and *p53* are required for irradiation-induced cell death and function as tumor suppressor genes by preventing the accumulation of cells harboring cancer-causing mutations. Since the identification of the *Drosophila* ortholog of the *p53* gene by whole genome sequencing^2,3^, *Drosophila* has been used as a model organism to study the DNA damage response^4^. However, most studies in *Drosophila* and the mammalian system, have been performed using mitotically dividing somatic cells, and the DNA damage response in germline cells, including germline stem cells (GSCs), is relatively less characterized.

*Drosophila* adult females have a pair of ovaries, each containing approximately 16 ovarioles. Each ovariole consists of a germarium and several egg chambers with different developmental stages, up to 14. There are 2–3 GSCs in a germarium. GSCs undergo asymmetric cell division to produce a daughter GSC and a cystoblast that will undergo 4 synchronous cell divisions with incomplete cytokinesis to generate 16-cell cysts. Among the 16 cells, one cell is fated to become an oocyte and others become nurse cells, supplying proteins and RNAs to the oocyte at the end of oogenesis. Normal oogenesis is affected by stress, such as poor nutrition, resulting in cell death during early and mid-oogenesis and slowing of the cell cycle^5^. Previously, we have shown that high-dose irradiation induces cell cycle arrest of cystoblasts, cell death at early (germarium region 2) and mid-stage (stages 7–10), and morphological defects^6^. Most of these defects are recovered within a week after irradiation and the GSCs appear to survive high-dose irradiation and are able to produce viable oocytes. Since *p53* is a major gene involved in irradiation-induced cell death in somatic cells, we aimed to investigate the function of *p53* in oogenesis after irradiation.

**Figure 1:**
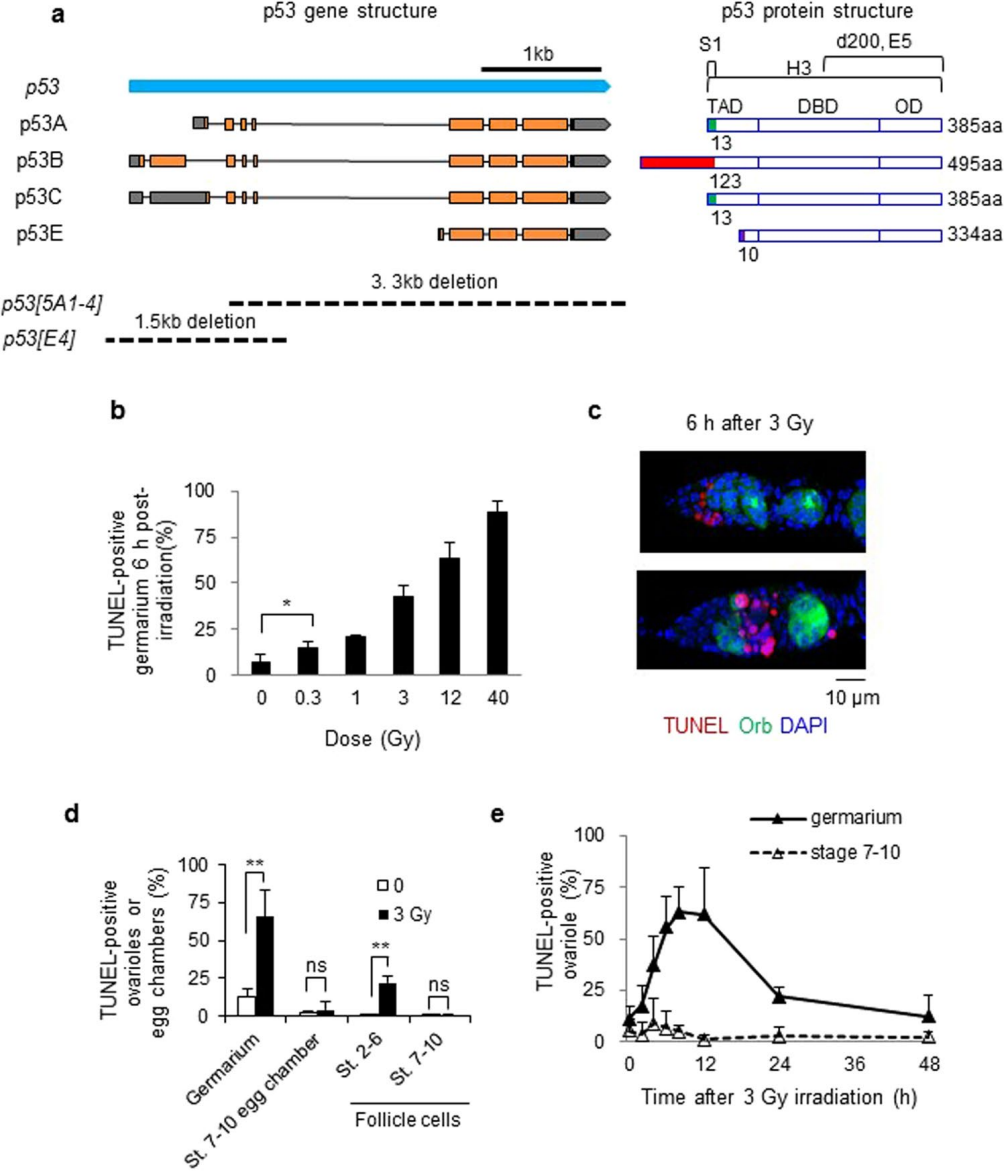
Cell death increases after irradiation in *Drosophila* ovaries. Wild type adult females were irradiated and dying cells in the ovary were detected by TUNEL staining. (**a**) Schema of the *Drosophila* *p53* mRNA and protein isoforms. Four *p53* RNA transcripts (left) predicted by the current *p53* gene annotation from Flybase are shown. Exons are indicated with boxes and the predicted open reading frames and 5′ or 3′ UTRs are indicated in orange and gray, respectively. The DNA regions deleted in *p53* mutants are shown below. The transcriptional transactivation domains (TAD), DNA binding domain (DBD), and oligomerization domain (OD) of each p53 isoform are shown (right). The N-terminal regions with unique amino acid sequences are indicated with different colors. The amino acid regions recognized by the p53 antibodies (S1, d200, E5, and H3) are indicated above. (**b**) The percentages of TUNEL-positive germarium were counted 6 h after irradiation at the indicated doses. The values are the mean ± standard deviation (SD) of three independent experiments (*p < 0.05). At least 192 ovarioles in total were counted for each dose. (**c**) Representative images of TUNEL-positive (red) germarium 6 h after 3 Gy irradiation are shown. DAPI (blue) and Orb (green) staining shows DNA and germline cells in the germarium region 2 and 3, respectively. (**d**) The percentage of ovariole containing TUNEL-positive germline cells in the germarium or egg chambers at stage 7–10 were counted. In the case of follicle cells, the egg chambers containing more than 4 TUNEL-positive follicle cells at stages 2–6 (St. 2–6) or at stages 7–10 (St. 7–10) were counted. The values are the mean ± SD of three independent experiments (ns *p* > 0.05, **p < 0.01). At least 290 ovarioles or 330 egg chambers for follicle cell death in total were counted. **(e)** Ovarioles containing TUNEL-positive germline cells in the germarium or stage 7–10 egg chambers were counted at 0, 2, 4, 6, 8, 12, 24, and 48 h after 3 Gy irradiation. The values are the mean ± SD of three independent experiments. At least 160 ovarioles in total were counted for each time point.

Historically, three different *Drosophila p53* isoforms, *p53A* (also reported as *dp53*, *p53*, *DΔNp53*, and *p53A*), *p53B* (*Dp53*, *p53B*), and *p53n* (*p53ΔC*), due to usage of alternative promoter and alternative splicing, were reported five years after the first isolation of the *Drosophila p53* gene, *p53A*^7^. Currently, four *p53* mRNA transcripts (A, B, C, and E) potentially encoding three different protein isoforms of 385, 495, and 334 amino acids (p53A, B, and E, respectively) are predicted in the *Drosophila* genome annotation^8^ (Fig. 1a). Except for the first 13, 123, and 10 amino acids of p53 A, B, and E, respectively, the amino acid sequences of the three isoforms are identical, generating a distinct N-terminal transactivation domain for each isoform with the same central DNA binding and C-terminal oligomerization domains (Fig. 1a).

Since the discovery of *p53* isoforms, various studies have been performed to identify the specific functions of each isoform. When the *p53A* and *p53B* isoforms are overexpressed at the same protein level in the larval disc cells by targeted integration of the transgene, both *p53A* and *p53B* induce apoptosis, while a smaller isoform, *p53E*, inhibits irradiation-induced apoptosis^9^. When a BAC clone specifically affecting each isoform was used for rescue analysis, *p53A*, but not *p53B*, was primarily required for DNA damage-induced apoptosis by inducing *hid* gene expression in the larval disc cells^9^. In addition to the mitotically dividing somatic cells mentioned above, the role of the *p53* isoforms in endocycling cells, where cell death is normally repressed, has been reported^10^. In this cell type, the overexpression of *p53B*, but not *p53A*, induces cell death. In male germline cells, *p53* is required for programmed necrosis which occurs in mitotic germ cells in the absence of stress^11^. On the other hand, relatively less is known about the role of *p53* isoforms in female germline cells.

In this study, we investigated irradiation-induced cell death during oogenesis and the role of *p53* isoforms in the *Drosophila* germline cells after low- and high-dose irradiation. We found that IR-induced cell death occurs at two developmental stages of oogenesis, depending on the irradiation doses. Using transgenic flies expressing *p53* isoforms, we found that the expression level of *p53A*, but not *p53B*, should be maintained at low levels to support normal oogenesis in the absence of stress. In response to DNA damage, both *p53* isoforms restored IR-induced apoptosis during early oogenesis. Moreover, the *p53A* rather than the *p53B* isoform was responsible for maintaining the function of GSCs after high-dose irradiation.

## Results

### Low-dose irradiation induces cell death during early oogenesis in *Drosophila* female

Previously, we have shown that high-dose irradiation induces rapid and massive germline cell death during early oogenesis followed by a slow and lower amount of cell death during mid-oogenesis^6^. To understand radiation-induced cell death during *Drosophila* oogenesis in greater detail, adult females were treated with different doses of IR. TUNEL-positive dying cells in the germarium region 2 increased in a dose-dependent manner (Fig. 1b,c). Cell death was slightly but significantly increased 6 h after irradiation at as low as 0.3 Gy (14.9% compared to 7.3% of untreated sample, *p* < 0.05). To facilitate detection, low-dose irradiation was performed after 3 Gy irradiation, which induced cell death in half of the germarium (Fig. 1b,e). These results were compared with those of high-dose irradiation at 40 Gy. The maximum level of cell death was detected in the germarium 8 h after 3 Gy irradiation and was reduced to basal levels 48 h after irradiation. Conversely, cell death was not increased in stage 7–10 egg chambers until 72 h after irradiation (Fig. 1e and data for 72 h not shown), at which time significant cell death was observed after 40 Gy^6^. Similar to high-dose irradiation^6^, 3 Gy irradiation induced cell death in mitotically dividing somatic follicle cells at stages 2–6, but not in endocycling follicle cells at stages 7–10 (Fig. 1d). Therefore, low-dose irradiation induced rapid cell death in early oogenesis and mitotically dividing follicle cells as with high-dose irradiation but did not induce delayed degeneration of mid-stage egg chambers, suggesting that early oogenesis is more sensitive to DNA damage than mid-oogenesis.

### *lok, p53*, and *hid* are required for IR-induced cell death in early oogenesis

**Figure 2:**
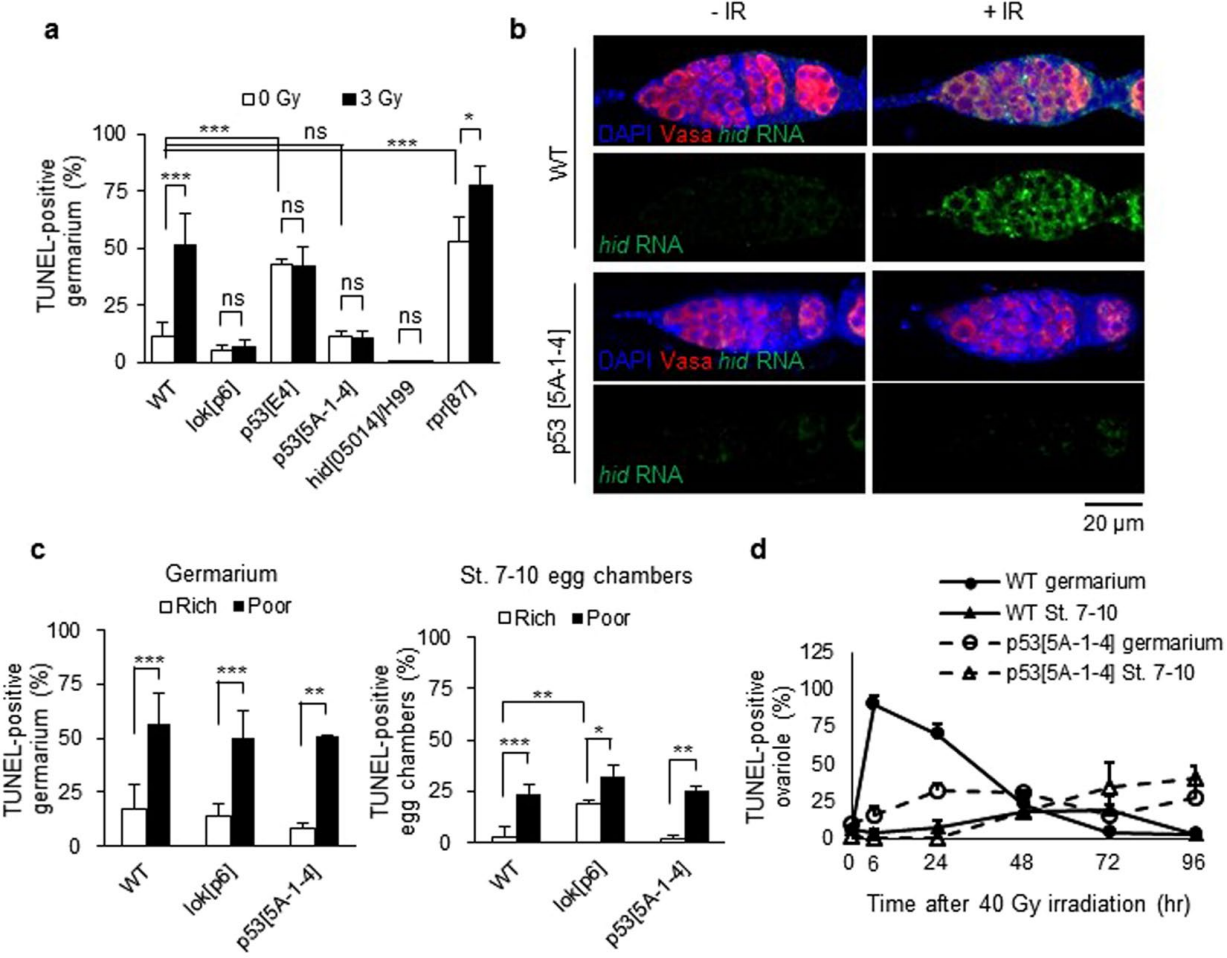
Genes involved in germline cell-death by IR and poor nutrition. (**a**) Females were irradiated at 3 Gy and TUNEL staining was performed with the ovaries 6 h after irradiation. The percentages of TUNEL-positive germline cells in the germarium region 2 in *lok*, *p53*, *hid*, and *rpr* mutant females were determined. The values are the mean ± SD of three independent experiments (ns *p* > 0.05, *p < 0.05, ***p < 0.001). At least 200 germarium in total were counted for each genotype. (**b**) Expression pattern of the *hid* transcript after irradiation. Wild type and *p53*^*5A-1-4*^ mutant females were irradiated at 40 Gy and RNA fluorescent *in situ* hybridization and protein immunofluorescence double labeling was performed using Dig-labeled anti-sense *hid* RNA as a probe and antibody against Vasa. (**c**) Wild type and mutant females were grown in rich or poor nutrition conditions and TUNEL staining of the ovaries was performed. The percentages of ovarioles containing TUNEL-positive germline cells in the germarium region 2 (left panel) and TUNEL-positive egg chambers of stage 7–10 are shown (right panel). The values are the mean ± SD of at least two independent experiments (*p < 0.05, **p < 0.01, ***p < 0.001). At least 235 ovarioles in total were counted for each genotype. (**d**) Wild type and *p53*^*5A-1-4*^ mutant females were irradiated with 40 Gy and ovarioles containing TUNEL-positive germline cells in the germarium region 2 or stage 7–10 egg chambers were counted at 6, 24, 48, 72, and 96 h after irradiation. The values are the mean ± SD of at least two independent experiments. At least 100 ovarioles in total were counted for each timepoint.

IR-induced apoptosis in *Drosophila* somatic cells requires the *Drosophila* ortholog of *Chk2 (lok)*^12^ and *p53 (p53)*^13^. Additionally, proapoptotic genes such as *reaper* and *hid*, which are induced by *p53*, are also required for cell death and the contribution of each gene depends on cell context. In our previous study, we investigated whether the same signaling pathway was induced in the germline cells and found that 40 Gy-induced cell death in the germarium required *p53* and *lok*^6^. To further develop this finding, we investigated the cell death in the mutant germarium 6 h after 3 Gy. Cell death was increased in the *reaper* mutant but not in the *lok*, *p53*, and *hid* mutants, suggesting that *lok*, *p53*, and *hid*, but not *reaper*, are necessary for low-dose IR-induced cell death in the germarium (Fig. 2a). We also found that *hid* is required for IR-induced cell death in the germarium when irradiated at 40 Gy (Fig. S1). Therefore, *lok*, *p53*, and *hid* are required for cell death of the germline cells in the germarium after both low- and high-dose irradiation. To test if *hid* transcription is induced by *p53* in the ovary after 40 Gy irradiation, RNA fluorescent *in situ* hybridization (FISH) and protein immunofluorescence (IF) double labeling was performed. When Dig-labeled anti-sense *hid* RNA was used as a probe, the *hid* transcript was increased in wild type germarium and egg chambers but not in *p53* mutant after irradiation (Figs 2b and S2a). The signal was not detected before and after irradiation with a sense probe (Fig. S2b). These results suggest that irradiation-induced cell death in the germarium occurs through *p53*-dependent transcriptional activation of the *hid* gene. It is interesting to note that the *hid* transcript is induced in the entire germarium, suggesting that post-transcriptional regulation of *hid* is necessary for region 2-specific cell death after irradiation.

Poor nutrition also induces cell death in early and mid-oogenesis and *p53* has been shown to be dispensable for cell death in mid-oogenesis^14^. To confirm and extend the genetic requirements of cell death induced by poor-nutrition, we tested whether cell death occurs in *lok* and *p53* mutant ovaries. In contrast to irradiation, starvation of *lok* and *p53* mutant females significantly increased cell death in the germarium (*p* < 0.001 and *p* < 0.01, respectively) and stage 7–10 egg chambers (*p* < 0.05 and *p* < 0.01, respectively) (Fig. 2c). This result suggests that different signaling pathways are utilized to induce cell death in the germarium depending on the nature of the stimuli: poor nutrition or DNA damage. We also found that the basal level of cell death in the germarium and stage 7–10 egg chambers was significantly increased in *p53*^*E4*^ (*p* < 0.001), *rpr*^87^ (*p* < 0.001), and *lok*^*p6*^ (*p* < 0.01), respectively. Increased cell death in the germarium in *p53*^*E4*^ does not appear to be induced by the lack of p53 protein since it was not observed in the null mutant, *p53*^*5A-1–4*^, that disrupts all isoforms (Figs 1a and 2a). The role of *rpr* and *lok* in early and mid-stage cell death remains to be studied.

In the mitotically dividing somatic cells in *Drosophila*, cell death induced by high-dose irradiation occurs in two phases^15^: *p53*-dependent rapid cell death at high levels followed by slow lower-level cell death that occurs in a *p53*-independent manner. To test whether the delayed cell death in the germline cells by high-dose irradiation is also *p53*-independent, time-course analysis of TUNEL staining was performed in the wild type and *p53*^*5A-1-4*^ mutant after 40 Gy irradiation. Similar to the finding in the somatic cells, the rapid high-level cell death that was observed in the wild type germarium at 6 h post-irradiation (Fig. 2d solid line with closed circle) was not detected, whereas low level delayed cell death at 24-48 h after irradiation was observed in the *p53*^*5A-1-4*^ mutant germarium (Fig. 2d dotted line with open circle). Additionally, the slow and low-level cell death at the stage 7-10 egg chambers that was detected only after high-dose irradiation still occurred in the *p53*^*5A-1-4*^ mutant (Fig. 2d dotted line with open triangle, *p* > 0.05 at 48 h and 72 h compared to wild type). These results suggest that three different signaling pathways are activated in the ovary during two developmental stages in response to high-dose irradiation to remove damaged germline cell: *p53*-dependent, rapid, and high-level cell death followed by *p53*-independent, slow, and lower-level death occurs in the germarium region 2 and *p53*-independent, slower, and lower-level death occurs at stage 7–10.

### Role of *p53* isoforms in IR-induced cell death

**Figure 3:**
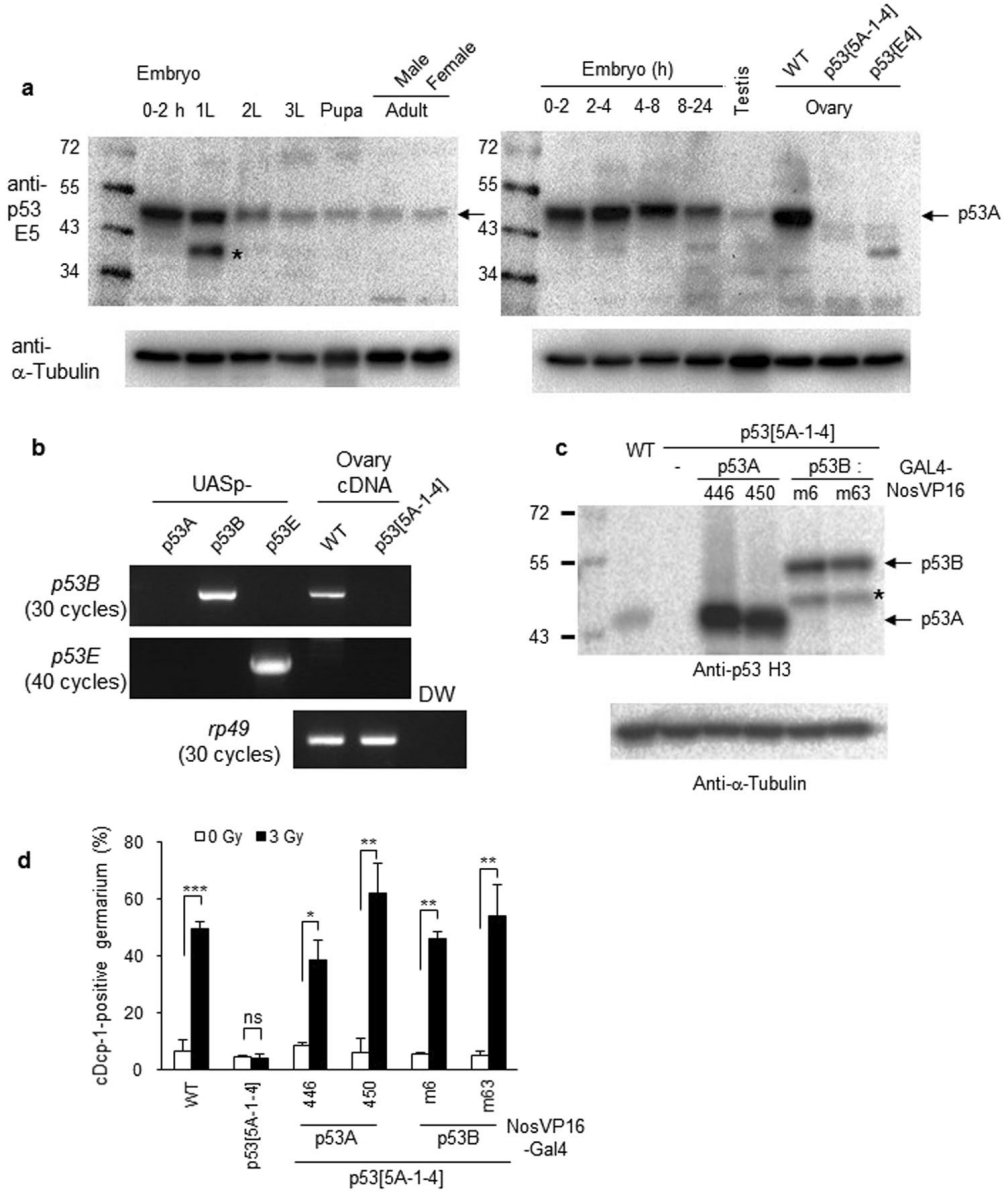
Expression of *p53A* and *p53B* can rescue IR-induced germline cell death in the *p53* mutant germarium. (**a**) Lysates were generated from the embryos of the indicated ages, first, second, third instar larvae (1 L, 2 L, and 3 L), pupae, adult males and females, and testis and ovaries from the wild type or *p53* mutants. The monoclonal antibody E5, which recognizes p53A, p53B and p53E was used. Bands corresponding to the p53A isoform are indicated by an arrow. Other bands, including the ones indicated by asterisks, appear to be a non-specific or degradation product of p53 protein. The membrane was stripped and reprobed with anti-α-Tubulin as a loading control. (**b**) To determine expression levels of the *p53B* and *p53E* transcripts, reverse-transcription PCR analysis was performed using total RNA preparation from ovaries of wild type and *p53* mutant flies. UASp constructs containing the cDNA of each *p53* isoform were used as controls. (**c**) Western blot of ovaries from wild type or transgenic flies expressing the indicated *p53* isoform in the *p53*^*5A-1-4*^ mutant. Two independent *UASp-p53* insertion lines (446 and 450 for *p53A*, m6 and m63 for *p53B*) were used for each isoform. The blot was probed with anti-p53 H3 antibody followed by anti-α-Tubulin antibody. The band corresponding to each isoform is indicated. A signal indicated with * may be a degradation product of p53B isoform. (**d**) Transgenic lines expressing each *p53* isoform in the *p53*^*5A-1-4*^ mutant germline cells were tested for IR-induced cell death in the germarium by staining with antibody against cleaved Dcp-1 (cDcp-1). The values are the mean ± SD of three independent experiments (ns *p* > 0.05, *p < 0.05, ***p < 0.001). At least 200 germariums in total were counted for each sample except for *p53A(450)* rescued flies (48 and 76 germariums for 0 Gy and 40 Gy, respectively). Uncropped gels and blots are shown in Supplementary Fig. S5.

Although the RNA-seq data suggests that the *p53B* mRNA is expressed throughout *Drosophila* development^8^, endogenous p53B protein has not been reported. To determine the expression pattern of various *p53* isoforms during development, we performed developmental western blotting using p53 E5 antibody, which recognizes the p53A, p53B, and p53E isoforms (Fig. 1a). The p53A isoform (with a molecular weight of 44 kDa) that was not detected in the *p53* mutant ovaries was observed throughout the developmental stages with relatively higher levels in the early embryo and the adult ovaries (Fig. 3a). On the other hand, no specific band corresponding to the p53B or p53E (with a molecular weight of 56 kDa and 38 kDa, respectively) isoforms was detected, suggesting that the p53A is the major isoform expressed throughout development. To determine whether the *p53B* and *p53E* isoforms are expressed in the ovary, RT-PCR analysis was performed with total RNA isolated from the wild type and *p53*^*5A-1-4*^ mutant ovaries. Using primers that specifically amplify each isoform (Fig. 3b lanes; 1, 2, and 3), the *p53B* transcript was detected in the wild type but not in the *p53*^*5A-1-4*^ mutant ovaries after 30 cycles of amplification (Fig. 3b, upper panel). On the other hand, the *p53E* transcript was not detected even after 40 cycles of amplification (Fig. 3b, middle panel).

Since *p53E* was not detected by RT-PCR in the ovary, we focused on the functions of the *p53A* and *p53B* isoforms during oogenesis after irradiation. After expressing each *p53* isoform in the ovary using germline-specific *GAL4* (*GAL4*-*NosVP16*) in *p53*^*5A-1-4*^ background, the expression level of each transgene from two independent transgenic lines was analyzed. Compared to the endogenous p53A protein in the ovary, the expression level of p53A and p53B transgene was approximately 14~23- and 5~6-fold higher, respectively (Fig. 3c). When p53A was expressed by *GAL4*-*NosVP16* in a *p53* mutant background and stained with antibody against Vasa, which detects germline cells, severe morphological defects were observed, most likely due to toxicity. We counted the cell death phenotype only in the germariums that showed an apparently normal Vasa staining pattern. In response to irradiation, lack of cell death in *p53*^*5A-1-4*^ mutant germarium was rescued by the expression of *p53A* and *p53B* (Fig. 3d), suggesting that both *p53A* and *p53B* isoforms can support IR-induced germline cell death in the germarium.

### Biochemical characterization of *Drosophila p53* isoforms

**Figure 4:**
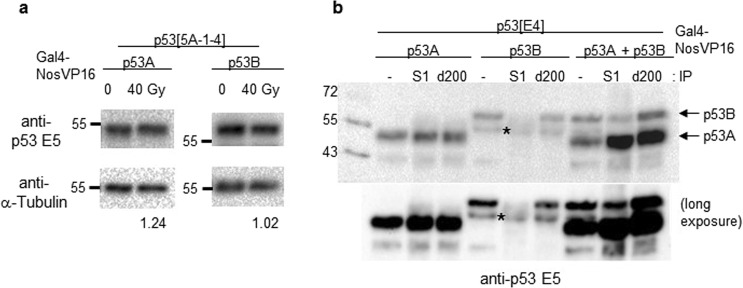
Biochemical characterization of p53 isoforms. (**a**) *p53* mutant flies expressing p53A or p53B isoforms in the germline cells were irradiated at 40 Gy. Ovary extracts were generated 1 h after irradiation and western blotting was performed. The representative images are shown. After normalizing to the loading control α -Tubulin, the p53 protein levels after irradiation is indicated below the blot by setting each p53 isoform level from non-irradiated control as 1. (**b**) p53A or p53B protein was expressed individually or together in the germline cells of *p53*^*E4*^ mutant using *GAL4-NosVP16* driver. The ovary extract was used to precipitate p53A isoform using the p53 S1 antibody, which recognizes p53A, not p53B. Immunoprecipitation with p53 d200 antibody, which recognizes both p53A and p53B, was used as a control. Western blotting was performed using the p53 E5 antibody. Lanes with “-“ indicate the input. The short and long exposures of the images are shown. Uncropped blots are shown in Supplementary Fig. S5.

In the mitotically dividing somatic cells, the mRNA and protein levels of *Drosophila p53* are increased after irradiation, but protein stability is not increased due to lack of the *MDM2* ortholog in *Drosophila*^10^. To test whether the p53A or p53B protein levels in the germline cells are changed after irradiation, we used *p53* mutant flies expressing the *p53A* or *p53B* isoform in the germline cells by *GAL4-NosVP16*. Neither the p53A nor p53B protein levels were markedly increased after irradiation (Fig. 4a), suggesting that irradiation does not induce *Drosophila* p53 protein stabilization in the germline cells.

In addition to protein stabilization, the tetramerization of p53 is also important for the transcriptional activation of p53^16^. Similar to human p53, the *Drosophila* p53 isoforms, both p53A and p53B, contain oligomerization domain, which has been shown to form tetramers^17^. To test whether p53A and p53B can form a heterotetramer, we developed a polyclonal antibody S1 that could specifically interact with p53A but not with p53B. We also generated *p53* mutant flies that could express either *p53A* or *p53B* alone or the two isoforms together using *GAL4-NosVP16* driver. Immunoprecipitation analysis of the ovary lysates confirmed that the p53A-specific antibody (S1) could precipitate p53A but not p53B (Fig. 4b lanes 2, and 5). When the S1 antibody was used to precipitate p53 in the flies expressing both p53A and p53B, the p53B isoforms were pulled down, confirming that p53A and p53B could form a complex (Fig. 4b lane 8).

### Role of *p53* isoforms in egg production of irradiated females

**Figure 5:**
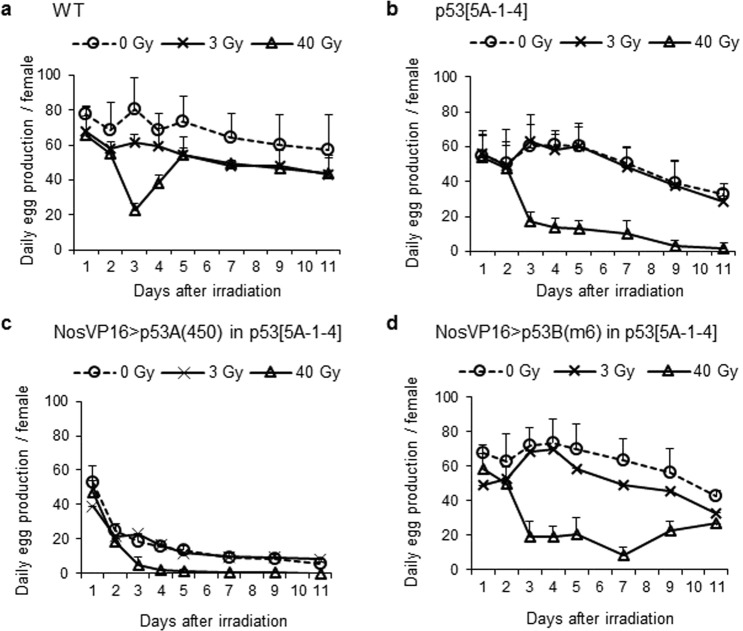
Role of p53 isoforms in the egg production of females after low- and high-dose irradiation. Females of the indicated genotypes (**a**: wild type, **b**: *p53*^*5A-1-4*^, **c**: *GAL4-NosVP16/UASp-p53A(450); p53*^*5A-1-4*^, **d**: *GAL4-NosVP16/UASp-p53B(m6); p53*^*5A-1-4*^) were irradiated at 0, 3, or 40 Gy and the eggs were collected every 24 h. Day 1 corresponds to the egg collection during the first 24 h after irradiation. The number of eggs per female were determined. The data are presented as the mean ± SD of the number of eggs per female of at least two independent experiments. Each experiment was performed using 5 cages for each treatment and each cage contained 3 pairs of females and wild type unirradiated males.

To test the role of *p53* isoforms during oogenesis after irradiation, we compared the egg production of females after 3 and 40 Gy irradiation. The wild type females irradiated at 3 Gy showed slightly reduced egg production compared to the non-irradiated females until 11 days after irradiation (Fig. 5a). Interestingly, the large reduction of egg production observed in the females between 3–4 days after 40 Gy irradiation was not detected in the 3 Gy-irradiated females. This may be due to cell death in stage 7–10 egg chambers, which was induced after irradiation at 40 Gy but not at 3 Gy. In the 3 Gy-irradiated *p53*^*5A-1-4*^ mutant females, daily egg production was not significantly different from that of the unirradiated females (*p* > 0.05) (Fig. 5b), suggesting that reduced egg production in the 3 Gy-irradiated wild type females may be due to *p53*-dependent cell death. After 40 Gy irradiation, the reduction in egg production 3 days after irradiation in the *p53*^*5A-1-4*^ mutant females was never recovered and continued to decrease (Fig. 5b). In the *p53*^*5A-1-4*^ mutant females expressing the *p53A* isoform by *Gal4-NosVP16*, we could not conclude whether *p53A* could restore the egg production after irradiation since the daily egg production was severely reduced even in the absence of irradiation (Fig. 5c). When *p53*^*5A-1-4*^ mutant females were rescued by the *NosVP16*-driven *p53B* isoform, daily egg production after 3 Gy-irradiation was slightly reduced compared to unirradiated female similar to wild type females (Fig. 5d). In response to 40 Gy irradiation, the egg production that was decreased 3 days after irradiation remained low until day 7, but gradually increased by day 11 (Fig. 5d). These results indicate that the *p53B* isoform can rescue the changes in egg production in *p53* mutant females after low- and high-dose irradiation.

### Moderate level expression of p53A and p53B isoform can restore loss of germline stem cells (GSCs) in the *p53* mutant after high-dose irradiation

**Figure 6:**
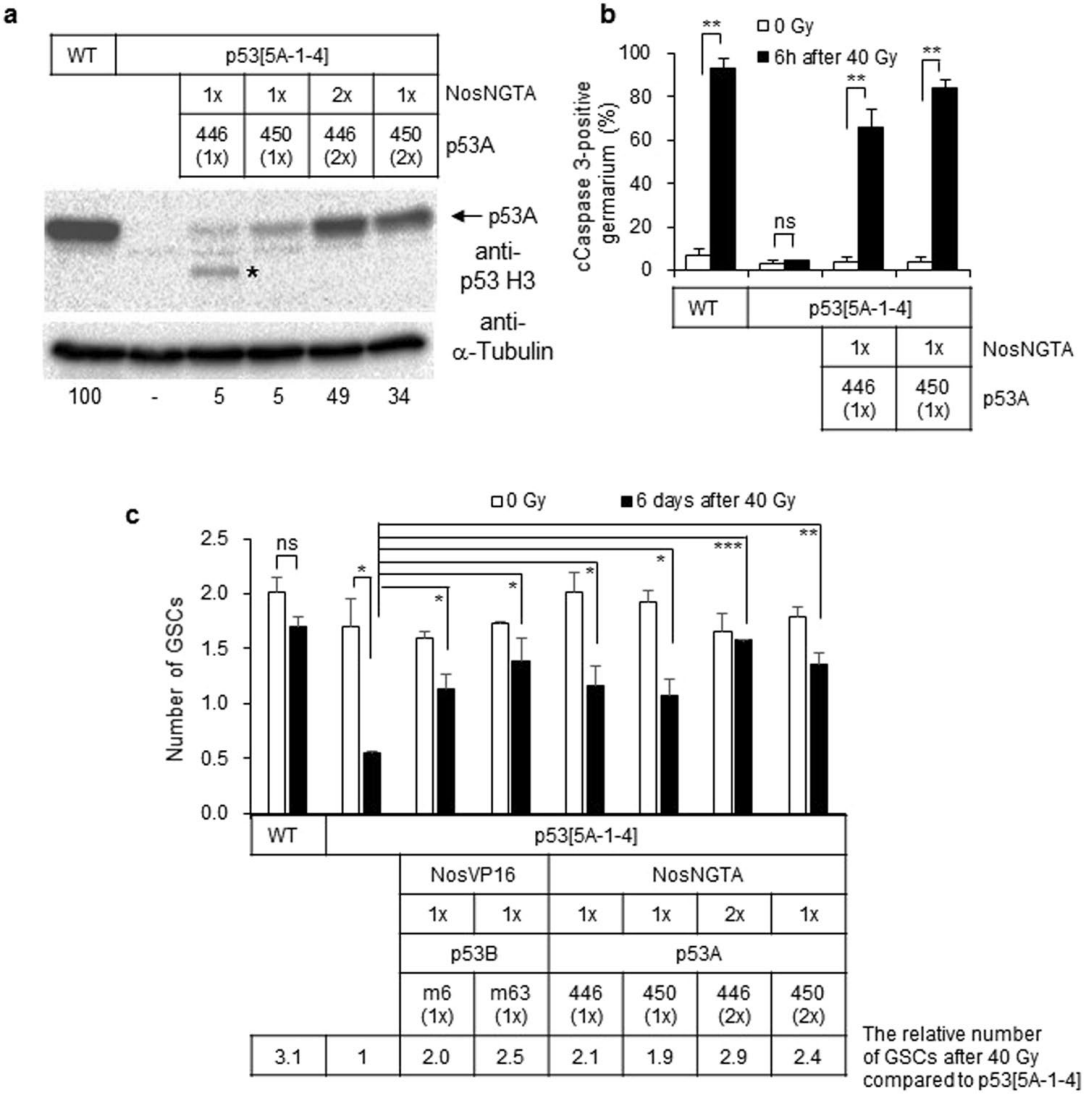
The moderate level expression of the p53A and p53B isoforms restores the loss of GSCs in the *p53*^*5A-1-4*^ mutant after high-dose irradiation. (**a**) A representative image of the western blotting of the ovaries from wild type, *p53*^*5A-1-4*^ mutant, and transgenic flies expressing *p53A* by *Gal4-NosNGTA* driver in *p53*^*5A-1-4*^ mutant background [lane 3: *UASp-p53A(446)*/+; *GAL4-NosNGTA, p53*^*5A-1-4*^/+, *p53*^*5A-1-4*^, lane 4: *UASp-p53A(450)*/+; *GAL4-NosNGTA, p53*^*5A-1-4*^/+, *p53*^*5A-1-4*^, lane 5: *UASp-p53A(446); GAL4-NosNGTA, p53*^*5A-1-4*^, and lane 6: *UASp-p53A(450); GAL4-NosNGTA, p53*^*5A-1-4*^/+, *p53*^*5A-1-4*^] is shown. The blot was probed with anti-p53 H3 antibody followed by anti-α-Tubulin antibody. The relative amount of p53A levels normalized by Tubulin is shown below the blot compared to that of wild type as 100. A signal indicated with * may be a degradation product of p53A isoform. (**b**) The wild type, *p53*^*5A-1-4*^ mutant, and transgenic flies expressing *p53A* by *Gal4-NosNGTA* driver in *p53*^*5A-1-4*^ mutant background were irradiated at 40 Gy and the germline cell death in the germarium was assayed by staining with antibodies against cleaved Caspase 3. The data represent the mean ± SD of two independent experiments (ns *p* > 0.05, **p < 0.01). At least 153 germariums in total were counted for each sample. (**c**) The wild type, *p53*^*5A-1-4*^ mutant, and *p53*^*5A-1-4*^ mutant flies expressing *p53A* or *p53B* were irradiated at 40 Gy. Six days later, the number of GSCs in the germarium was analyzed by staining with 1B1, Vasa, and Lamin C to detect fusome, germline cells, and cap cells, respectively. The values are the mean ± SD of two independent experiments (ns *p* > 0.05, *p < 0.05, **p < 0.01, ***p < 0.001). At least 51 germariums in total were counted for each sample. The transgenes were used as hemizygous (1×) or homozygous (2×) state. The number of GSCs after irradiation in each genotype compared to that of *p53*^*5A-1-4*^ mutant is indicated below by setting the number of GSCs in irradiated *p53*^*5A-1-4*^ mutant as 1. Uncropped blots are shown in Supplementary Fig. S5.

Since high levels of *p53A* isoforms were found to affect normal oogenesis, we attempted to reduce the expression level using another germline-specific driver, *GAL4-NosNGTA*. By altering transgene dosages, the expression level of the p53A isoform in the *p53* mutant background was reduced to 5–49% of the endogenous p53A levels in the wild type ovary (Fig. 6a). Even though the expression level of p53A was reduced to 5% of that of endogenous p53A, *NosNGTA*-driven p53A was functional because it was able to restore the lack of cell death 6 h after irradiation in the germarium (Fig. 6b).

To confirm whether the changes in egg production represent loss of GSCs, we counted the number of GSCs after high-dose irradiation. The GSCs were identified as a germline cells at a close vicinity to the cap cell and were found to contain round fusomes by staining with VASA (germline cells), 1B1 (fusome), and Lamin C (cap cells) antibodies. In the absence of irradiation, the number of GSCs in the *p53*^*5A-1-4*^ mutant, and the *p53*^*5A-1-4*^ mutant expressing *p53A* or *p53B* was not significantly different from that of the wild type (Fig. 6c). Six days after 40 Gy irradiation, the number of GSCs in the wild type germarium was not significantly different from the untreated control (0 Gy: 2.0 vs 40 Gy: 1.7, Fig. 6c). On the other hand, the number of GSCs in the *p53*^*5A-1-4*^ mutant was significantly decreased from 1.7 to 0.6 at 6 days after irradiation (*p* < 0.05) suggesting that reduced egg production in the irradiated *p53*^*5A-1-4*^ mutant females is due to the loss of GSCs. To test which *p53* isoform is involved in this process, we used two transgenic lines expressing *p53B* by *GAL4-NosVP16* and four transgenic lines expressing *p53A* by *GAL4-NosNTGA*. The number of GSCs after irradiation in these flies were 1.9–2.9-fold higher (3.1-fold higher in wild type) than that of the *p53*^*5A-1-4*^ mutant, suggesting that both *p53A* and *p53B* can partially rescue loss of GSCs after irradiation when expressed at moderate levels. It is interesting to note that the number of GSCs in the irradiated wild type and the *p53*^*5A-1-4*^ mutant expressing *p53B* were restored to untreated level by day 6 after a slight reduction at 3 days post-irradiation (Fig. S3). On the other hand, the number of GSCs in the *p53*^*5A-1-4*^ mutant continuously decreased by day 6 (Fig. S3), suggesting that *p53* may be required for the self-renewal of GSCs.

### Low level expression of *p53A* but not *p53B* isoform can restore loss of fertility in the *p53* mutant after high-dose irradiation

**Figure 7:**
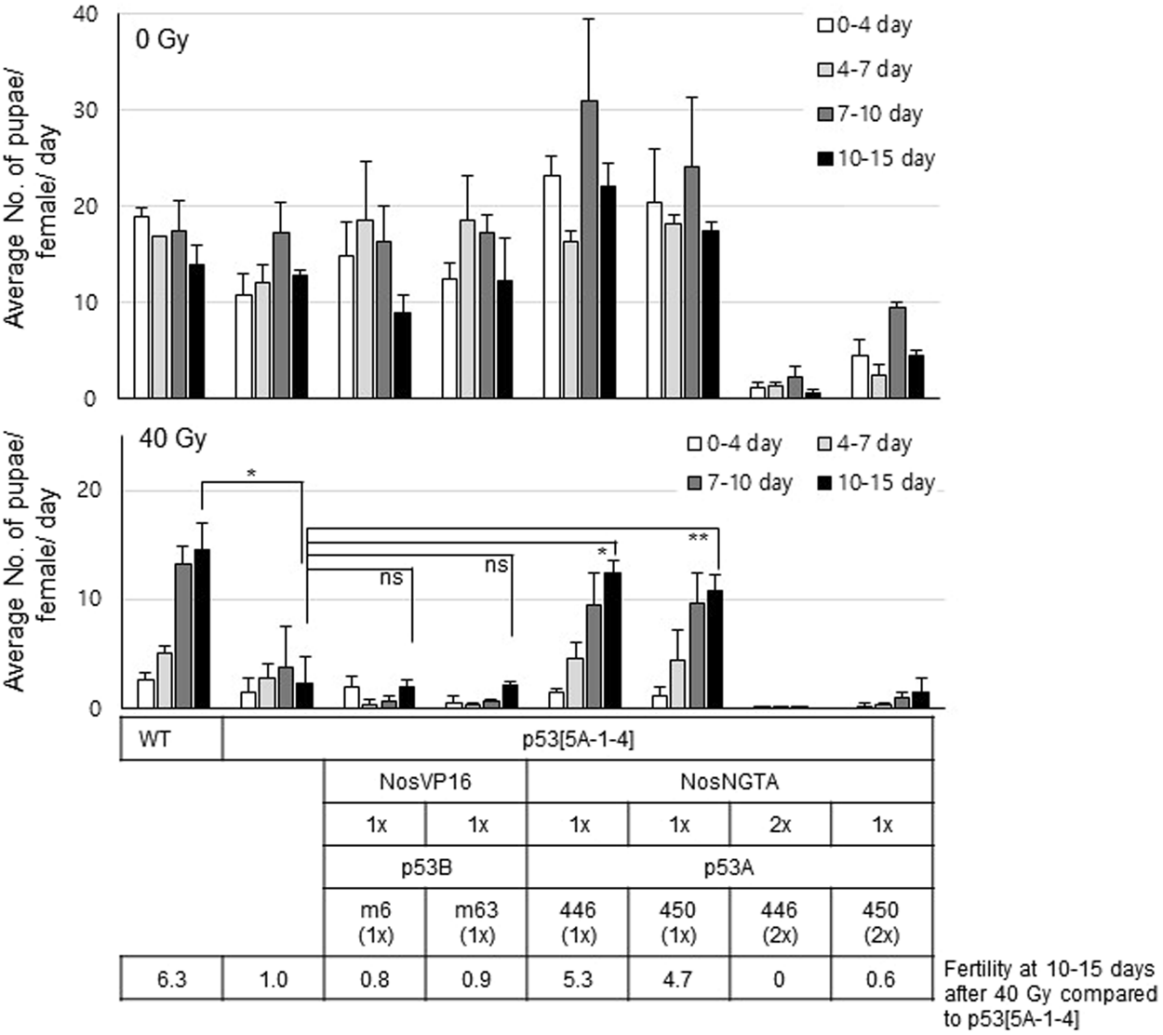
The expression of the *p53A* isoform at low levels restores the loss of fertility in the *p53*^*5A-1-4*^ mutant after high-dose irradiation. The adult female flies of the indicated genotypes were irradiated at 0 or 40 Gy and incubated with non-irradiated males. The adults were transferred to new vials every 24 h. The fertility was scored by the number of pupae produced 7–8 days after the adults were removed. The data represent the mean ± SD of the number of pupae produced per day by a single female during the indicated period after irradiation of at least two independent experiments (ns *p* > 0.05, *p < 0.05, **p < 0.01). The transgenes were used as hemizygous (1×) or homozygous (2×) state. The fertility after irradiation at 10–15 days in each genotype compared to that of *p53*^*5A-1-4*^ mutant is indicated below by setting the fertility of irradiated *p53*^*5A-1-4*^ mutant as 1.

To test whether the GSCs detected after high-dose irradiation were functional, the fertility of the adult female flies was tested by the production of pupae from the eggs laid by the female. In the absence of irradiation, the fertility of the wild type and *p53*^*5A-1-4*^ mutant females was not significantly different at days 10-15 (Fig. 7 upper panel). The fertility of the irradiated wild type females was very low during the first 4 days after irradiation; however, it gradually increased up to that of the non-irradiated control at 10–15 days after irradiation (Fig. 7 lower panel). When *p53*^*5A-1-4*^ mutant females were irradiated, the fertility was not increased at 4–15 days and remained low supporting the previous findings that high-dose irradiation reduces the egg production and the number of GSCs in the *p53*^*5A-1-4*^ mutant (Figs 5b and 6c).

In the case of the *p53*^*5A-1-4*^ mutant expressing *p53A* more than 30% of endogenous p53A, the fertility in the absence of irradiation was severely lower than that of the wild type (Fig. 7 last two genotypes in the upper panel). This result suggests that the GSCs in these flies are not functional and the amount of p53A protein should be maintained at low levels to support normal oogenesis. The expression of p53A at 5% of the endogenous p53A levels was able to support normal oogenesis in the absence of irradiation and could partially restore the fertility of the *p53*^*5A-1-4*^ mutant female after irradiation (4.7 and 5.3-fold higher than *p53*^*5A-1-4*^ mutant compared to 6.3-fold higher in the wild type, Fig. 7 5^th^ and 6^th^ genotypes). However, expression of *p53B*, which showed normal oogenesis without stress, was not able to restore the fertility (0.8- and 0.9-fold less than *p53*^*5A-1-4*^ mutant, Fig. 7). These results suggest that both *p53A* and *p53B* isoforms could maintain the number of GSCs after high-dose irradiation, but the GSCs maintained by *p53A*, not by *p53B*, was functional to produce progeny.

## Discussion

To understand the role of *p53* isoforms in germline cells, we investigated the cellular response of germline cells during *Drosophila* oogenesis after low- and high-dose irradiation. We found that IR-induced apoptosis occurs at two developmental stages of oogenesis through different mechanisms. Rapid and high-level apoptosis is induced during early oogenesis in the germarium region 2 involving *lok*, *p53A*, *p53B*, and *hid* in response to both low- and high-dose irradiation. Mid-oogenesis was relatively resistant to irradiation and slow and low-level cell death was induced in a *p53*-independent manner only after high-dose irradiation. The expression levels of the *p53* isoforms have distinct effects on oogenesis in the absence and presence of DNA damage. First, the expression level of *p53A*, but not *p53B*, should be maintained at a low level to support normal oogenesis in the absence of stress. Second, both *p53* isoforms were able to induce IR-induced apoptosis during early oogenesis. Third, both *p53A* and *p53B* isoforms could restore the number of GSCs after high-dose irradiation. Lastly, the *p53A* rather than the *p53B* isoform was responsible for maintaining the function of GSCs after high-dose irradiation.

During normal development, cell death occurs in nurse cells at the late-stage of oogenesis (stages 12–14). Additionally, cell death can be induced in a stage-specific manner at early- and mid-oogenesis in response to developmental abnormalities or environmental stress such as poor nutrition^18^. We found that irradiation also induces death in germline cells in early- and mid-stage oogenesis. Cell death in the germarium occurs through apoptosis since both TUNEL and cleaved Dcp-1 positive cells were increased after irradiation (Figs 1b and 3d) and TUNEL and cleaved Dcp-1-positive cells appeared in close vicinity (Fig. S4). Cell death after irradiation in the germarium required *lok* and *p53*, while these genes were not necessary for poor nutrition-induced cell death (Fig. 2a,c). Irradiation-induced cell death in the germarium also requires *hid*, but not *reaper*, and *hid* transcription is induced by irradiation in a *p53*-dependent manner throughout the germarium (Fig. 2b) although cell death occurs only in region 2 (Fig. 1c). This is not surprising since *hid* is expressed both in cells that survive as well as in only a subset of cells that die^19^, suggesting that *hid* may be negatively regulated post-transcriptionally in the surviving cells. In support of this hypothesis, Hid is negatively regulated by EGFR signaling through MAPK-dependent phosphorylation^20^. The signaling pathway of cell death in mid-oogenesis by poor nutrition is unique in that it requires effector caspase *Dcp-1* but not proapoptotic genes nor *p53*^18^. We also found that high-dose irradiation-induced cell death in mid-oogenesis does not require *p53* (Fig. 2d). These results indicate that at least three distinct signaling pathways induce cell death in female germline cells in response to stress at two developmental stages: *lok*- and *p53*-dependent cell death in the germarium after irradiation, *lok*- and *p53*-independent cell death in the germarium in response to poor nutrition, and *p53*-independent cell death in mid-oogenesis in response to both irradiation and poor nutrition.

In response to DNA damage, the GSCs of adult *Drosophila* females immediately arrest the cell cycle and repair the damaged DNA faster than their differentiated progeny^21^. DNA damage-induced apoptotic cell death in the GSCs is prevented by the repression of *hid* transcription through the receptor tyrosine kinase Tie-mediated signaling pathway activated by the survival signal from dying daughter cells^22^. More severe DNA damage results in the loss of the GSCs and a progeny differentiation defect in a *lok*-dependent manner^21^. *Drosophila ATM*, *ATR*, and *p53*, but not *Chk1*, are required for preventing DNA damage-induced loss of GSCs^21^. We found that the *Drosophila p53* isoforms exhibit two functions in the GSCs after irradiation: both *p53A* and *p53B* were able to restore the number of GSCs by 6 days after high-dose irradiation (Fig. 6c). Interestingly, irradiated GSCs maintained by *p53A* expression, but not by *p53B*, were functional, as observed in irradiated females with restored fertility (Fig. 7). Since it takes approximately 10 days for the GSCs to develop into mature eggs and oogenesis does not undergo significant delay after irradiation^6^, the fertility of the irradiated females 10–15 days after irradiation is likely to represent the function of the irradiated GSCs. The mechanism of GSC maintenance by *p53* has not yet been fully elucidated, despite previous reports providing some clues. DNA repair does not appear to be responsible since the repair kinetics determined by phosphorylated histone H2Av staining were similar in the wild type and *p53* mutant GSCs^23^. Delay in entry into the cell cycle in the irradiated *p53* mutant GSCs was observed^23^, suggesting that the reduced self-renewal may result in the loss of GSCs. In support of this, self-renewal of irradiated human *p53*-deficient hematopoietic stem cells has been shown to be compromised in a serial transplantation experiment^24^. Since the number and function of GSCs were found to be affected in the *p53* mutant, *p53* may be required for the proper differentiation of stem cells. In addition, *p53* reporter activity is increased in GSCs after irradiation^23^, suggesting that the identification of *p53* target genes in irradiated GSCs could facilitate our understanding of the mechanism of *p53* in GSCs maintenance after severe DNA damage.

Fluorescence correlation spectroscopy analysis showed that approximately 60% of p53 proteins are in dimeric form in living cells and DNA damage induces rapid assembly into tetramers^16^. Even when the p53 level was decreased by translation inhibition, *p53* target genes were induced after DNA damage suggesting that the tetramerization in the absence of increased total p53 protein levels was sufficient to activate transcription^16^. We found that the *Drosophila* p53A and p53B proteins overexpressed in the germline cells are not stabilized after irradiation (Fig. 4a) and that they form a complex (Fig. 4b). These findings suggest that tetramerization may be the major mechanism of p53 activation in *Drosophila* and the relative protein levels of p53A and p53B will affect the relative levels of hetero- and homo-tetramers and resultant target gene expression. The identification of the oligomer-specific target genes will provide insights into the novel functions of *p53 in vivo*.

In summary, we have shown that early oogenesis is very sensitive to irradiation and induces cell death in a *p53A*- and *p53B*-dependent manner, whereas low-level apoptosis is induced in mid-oogenesis only after high-dose irradiation in a *p53*-independent manner. Moreover, the *p53A* rather than the *p53B* isoform is responsible for maintaining the function of GSCs after high-dose irradiation. The apoptotic function of *p53* in early germline cells is similar to that of somatic cells, while the *p53A* isoform shows a unique function in adult female GSCs. The *Drosophila* ovary will serve as a great model system to further elucidate the novel functions of *p53* in stem cell biology.

## Materials and Methods

### *Drosophila* strains

All *Drosophila* strains were raised at 25 °C. *Canton S* or *w*^1118^ were used as wild type controls. *Drosophila* strains were obtained from the Bloomington *Drosophila* Stock Center (BDSC; Bloomington, IN, USA) unless otherwise indicated. The *lok*^*p6*^, *p53*^*E4*^, *hid*^05014^, and *rpr*^87^ flies were provided by Drs. W. Theurkauf^25^, J. Chung^13^, H. Steller^19^, and K. White^26^, respectively. To generate flies expressing *p53* isoforms, the cDNA for each isoform was cloned into a *UASp* vector and the transgenic flies were generated by germline mediated P-element transformation.

### Sample preparation for immunoprecipitation and western blotting

Tissues from *Drosophila* at adult or various developmental stages were homogenized with buffer containing 50 mM Tris-HCl pH 7.4, 0.5% Nonidet P-40, 150 mM NaCl, 1 mM EDTA, 50 mM NaF, 20 mM NaVO_3_, 20 mM beta-glycerophosphate (Sigma-Aldrich; St. Louis, MO, USA), Protease Inhibitor Cocktail (Sigma-Aldrich), and 2 mM PMSF (Amresco; Solon, OH, USA). After centrifugation, the supernatants were used for immunoprecipitation or western blotting analysis following the standard procedures.

### Immunofluorescence staining and antibodies

Adult females were either mock-treated or irradiated in a Cs^137^ gamma-irradiator and the ovaries were then subjected to immunofluorescence and TUNEL staining as previously described^6^. For nutrition study, two to three-day old females were incubated with rich or poor food for two days and the ovaries were dissected for staining. Plain molasses plates were used as a poor food source and molasses plates containing a layer of wet yeast served as a rich food source^5^. A polyclonal antibody (S1) that could recognize p53A but not p53B was generated by immunizing rabbits with a synthetic polypeptide (MYISQPMSWHKESTD) in the N-terminal region of p53A. Peptide synthesis and antibody generation were performed at AbFrontier (Seoul, Korea). Other primary antibodies used in this study included p53 H3 (Developmental Studies Hybridoma Bank; Iowa City, IA, USA), p53 E5 (Santa Cruz; Dallas, USA), p53 d200 (Santa Cruz), Vasa (Santa Cruz), Lamin C (Developmental Studies Hybridoma Bank), 1B1 (Developmental Studies Hybridoma Bank) and α-Tubulin (Sigma-Aldrich). The following secondary antibodies were used: goat anti-mouse Alexa568 (1:400, Molecular Probes; Eugene, OR, USA), goat anti-mouse Alexa647 (1:200, Molecular Probes), goat anti-rabbit Alexa488 (1:200, Molecular Probes), goat anti-rabbit Alexa568 (1:400, Molecular Probes) and donkey anti-goat Alexa488 (1:200, Molecular Probes). The ovaries were visualized using a confocal laser scanning microscope (LSM 700, Carl Zeiss, Oberkochen, Germany) or a fluorescence microscope (IX71, Olympus; Tokyo, Japan).

### RNA fluorescent *in situ* hybridization (FISH) and protein immunofluorescence (IF) double labeling and RT-PCR

To generate RNA probes, part of the *hid* cDNA (375 bp) was cloned into pGEM-T easy vector (Promega; Madison, WI, USA) in sense and antisense orientation. The DNA plasmids were linearized by NdeI digestion and transcribed by T7 RNA polymerase using Digoxigenin (Dig) RNA labeling Mix (Roche Diagnostics; Germany) for 2 h at 37 °C. The reaction was stopped by adding 0.2 M EDTA (pH 8.0) and the RNA was precipitated by ethanol and dissolved in RNase free distilled water.

FISH/IF double labeling to detect the *hid* transcript and Vasa was performed as previously described with slight modifications^27^. Briefly, four- to six-day old females were irradiated at 40 Gy and ovaries were dissected in 1X PBS after 3–4 h. Samples were fixed in a 1:1 mixture of 4% formaldehyde (Sigma-Aldrich) and heptane (Sigma-Aldrich) for 20 min with rocking. Samples were permeabilized with 3 μg/ml Proteinase K for 13 min at room temperature and transferred to ice for 1 h before washing with 2 mg/ml glycine solution. After re-fixation and blocking, the samples were hybridized with 50 ng Dig-labeled RNA probe at 56 °C overnight. The ovaries were incubated with biotin conjugated mouse monoclonal anti-DIG (Jackson ImmunoResearch lab Inc., 1:400) and rat anti-Vasa (Developmental Studies Hybridoma Bank, 1:20) for 2 h at room temperature with shaking. Secondary antibody incubation was performed for 1 h with 1X streptavidin-HRP (Invitrogen) and goat anti-rat rhodamine (Molecular Probes, 1:200) at room temperature. Signal amplification was performed with Alexa 488 Tyramide conjugate (1:50) in 1X reaction buffer supplied with Alexa Flour^TM^ 488 Tyramide SuperBoost^TM^ kit (Invitrogen) for 1 h at room temperature. The reaction was terminated using stop solution for 10 min. The ovaries were mounted in 0.5% n-propyl gallate dissolved in glycerol.

RNA isolation from the ovary and the RT-PCR was performed as previously described^28^.

### Fertility test

Four- to five-day-old adult females were irradiated at 0 or 40 Gy. Three vials containing four irradiated females and four unirradiated wild type males per vial were prepared for each genotype. The flies were transferred to new vials each day for 15 days. Fertility was scored according to the presence of pupae 7 to 8 days after the parents were removed from the vials. The daily egg production was determined as previously reported^6^.

### Statistical analysis

The experiments were performed at least two times. The quantitative data are expressed as the mean ± SD. The significance of differences between two experimental samples was determined using two‐sided, unpaired Student’s *t*‐test. Differences were considered statistically significant at *p* < 0.05.

## Supplementary information

Role of p53 isoforms in the DNA damage response during Drosophila oogenesis

## Data Availability

All data generated during this study are included in this published article. Additional raw data will be available on request.
