# Supplementary material for: Role of p53 isoforms in the DNA damage response during Drosophila oogenesis
Source: Sci Rep. 2019 Aug 7;9:11473. doi: 10.1038/s41598-019-47913-y (PMC6685966; doi:10.1038/s41598-019-47913-y)
Supplement: Supplementary file 1 — Role of p53 isoforms in the DNA damage response during Drosophila oogenesis [file 41598_2019_47913_MOESM1_ESM.pdf]

Role of *p53* isoforms in the DNA damage response during *Drosophila* oogenesis

Ji-Hong Park[1§], Tram Thi Ngoc Nguyen[1§], Eun-Mi Lee[2], Veronica Castro-Aceituno[2], Ram Wagle[1], Kwang-Soon Lee[2], Juyoung Choi[2], and Young-Han Song[1,2*]

[1]Department of Biomedical Gerontology, Hallym University, Chuncheon, Gangwon-do, Republic of Korea

[2]Ilsong Institute of Life Science, Hallym University, Anyang, Gyeonggi-do, Republic of Korea,

***Corresponding author:** Young-Han Song. Telephone: 82-31-380-1897, Fax: 82-31-388-3427, E-mail: ysong@hallym.ac.kr

[§]These authors contributed equally.

Fig. S1

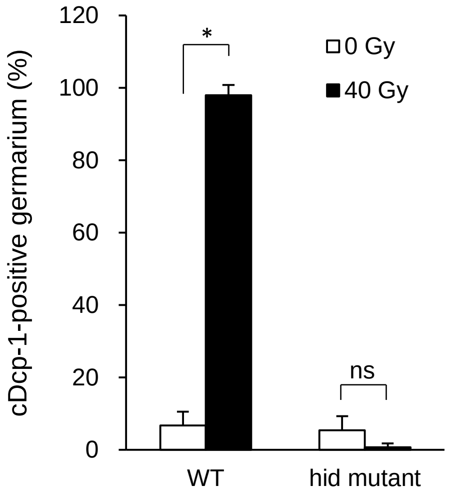

Fig. S1 The wild type and *hid* mutant (*hid[05014]*/*Df(3L)H99*) females were irradiated at 40 Gy and cleaved Dcp-1 (cDcp-1) staining was performed with the ovaries 6 h after irradiation. The percentages of cDcp-1-positive germline cells in the germarium region 2 were determined. The values are the mean ± SD of two independent experiments (ns $p > 0.05$, * $p < 0.05$). At least 118 germarium in total were counted for each sample.

Fig. S2

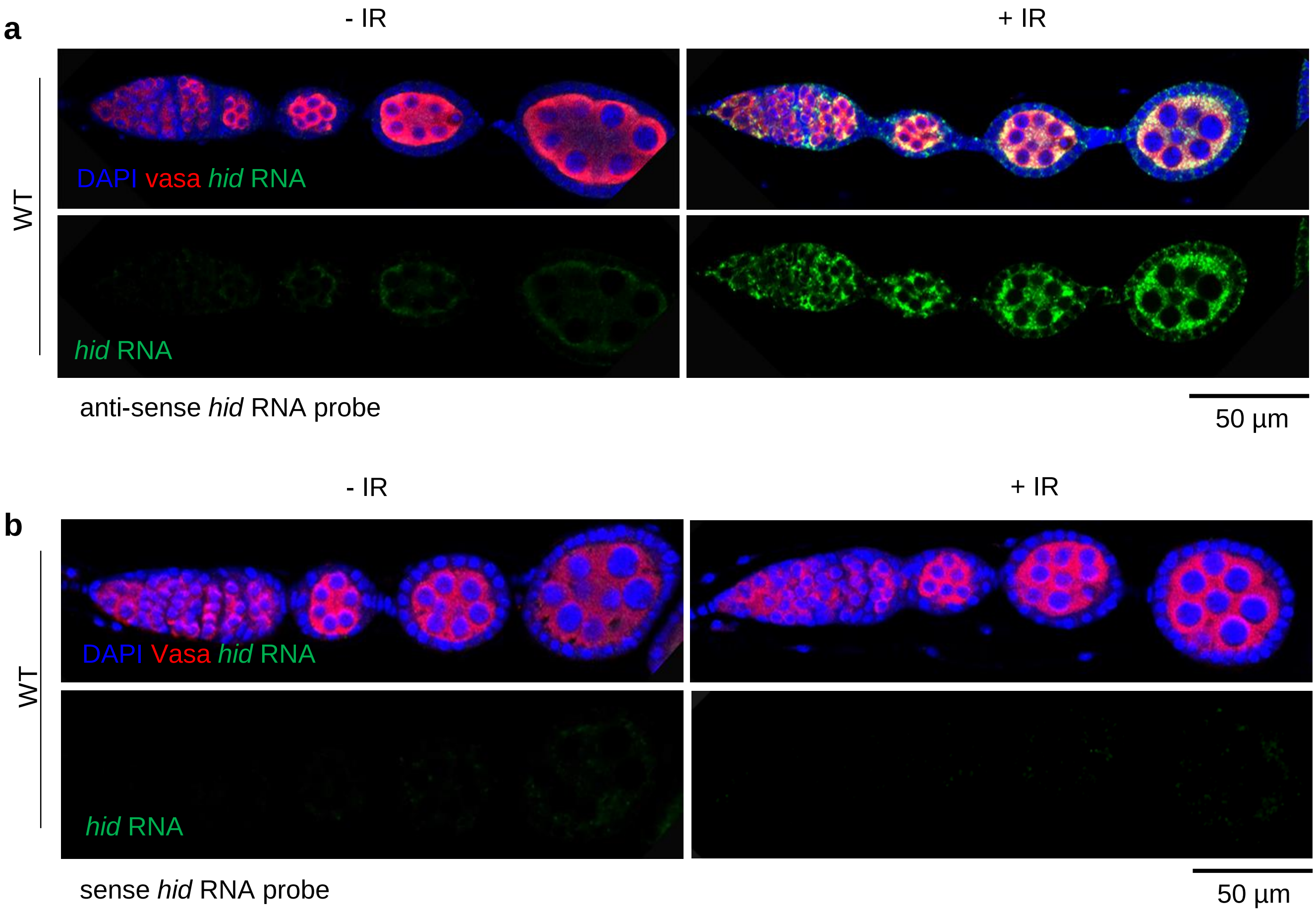

Fig. S2 Expression pattern of the *hid* transcript after irradiation. Wild type females were irradiated at 40 Gy and after 3-4 h RNA fluorescent *in situ* hybridization and protein immunofluorescence double labeling was performed using Dig-labeled anti-sense (**a**) or sense (**b**) *hid* RNA as a probe and antibody against Vasa.

Fig. S3

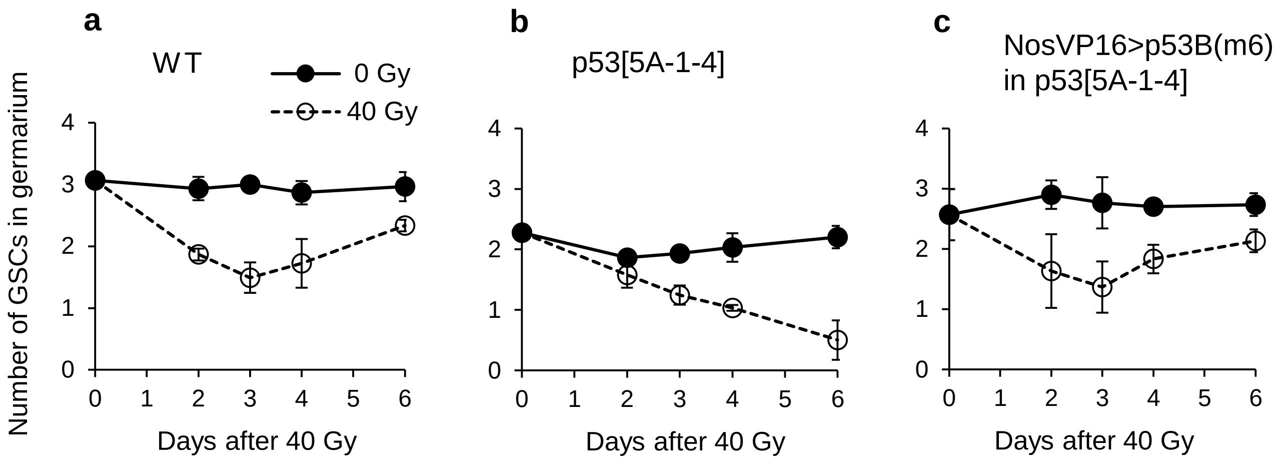

Fig. S3. The number of germline stem cells after irradiation in wild type and p53 mutant females. Females of indicated genotype (a: wild type, b: *p53*$^{5A\text{-}1\text{-}4}$, c: *GAL4-NosVP16/UASp-p53B; p53*$^{5A\text{-}1\text{-}4}$) were irradiated at 0 or 40 Gy and the ovaries were dissected and stained with 1B1, Vasa, and Lamin C to detect fusome, germline cells, and cap cells, respectively. The average number of germline stem cells in the germarium was determined. Values are mean ± SD for at least two independent experiments. At least 28 germarium in total were counted for each sample.

Fig. S4

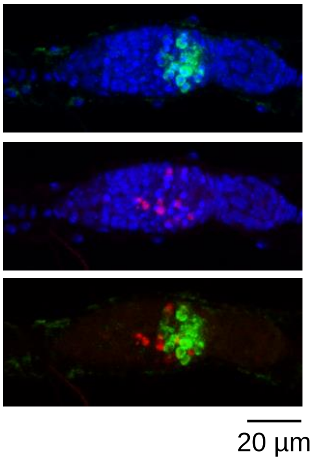

Fig. S4 Wild type adult females were irradiated at 3 Gy and dying cells in the ovary were detected by TUNEL and cleaved Dcp-1 (cDcp-1) double staining. Representative images of TUNEL (red) and cDcp-1 (green)-positive germarium 6 h after 3 Gy irradiation are shown. DAPI (blue) staining shows DNA.

Fig. S5. Uncropped images of western blots of Fig. 3a.

Fig. 3a

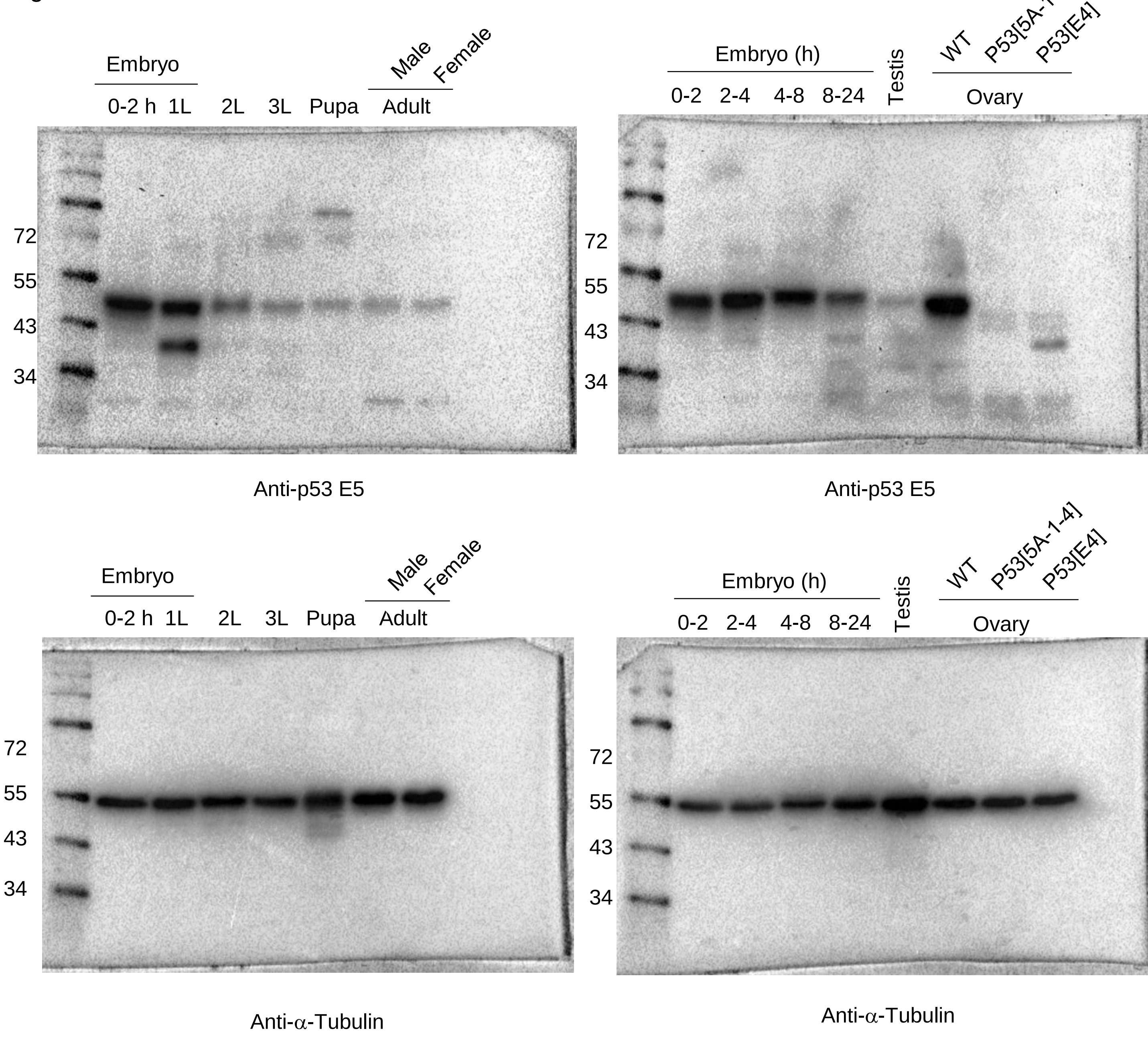

The membrane immunoblotted with anti-p53 antibody was stripped and reprobed with anti-α-Tubulin antibody.

Fig. S5. Uncropped images of western blots of Fig. 3b and 3c.

Fig. 3b

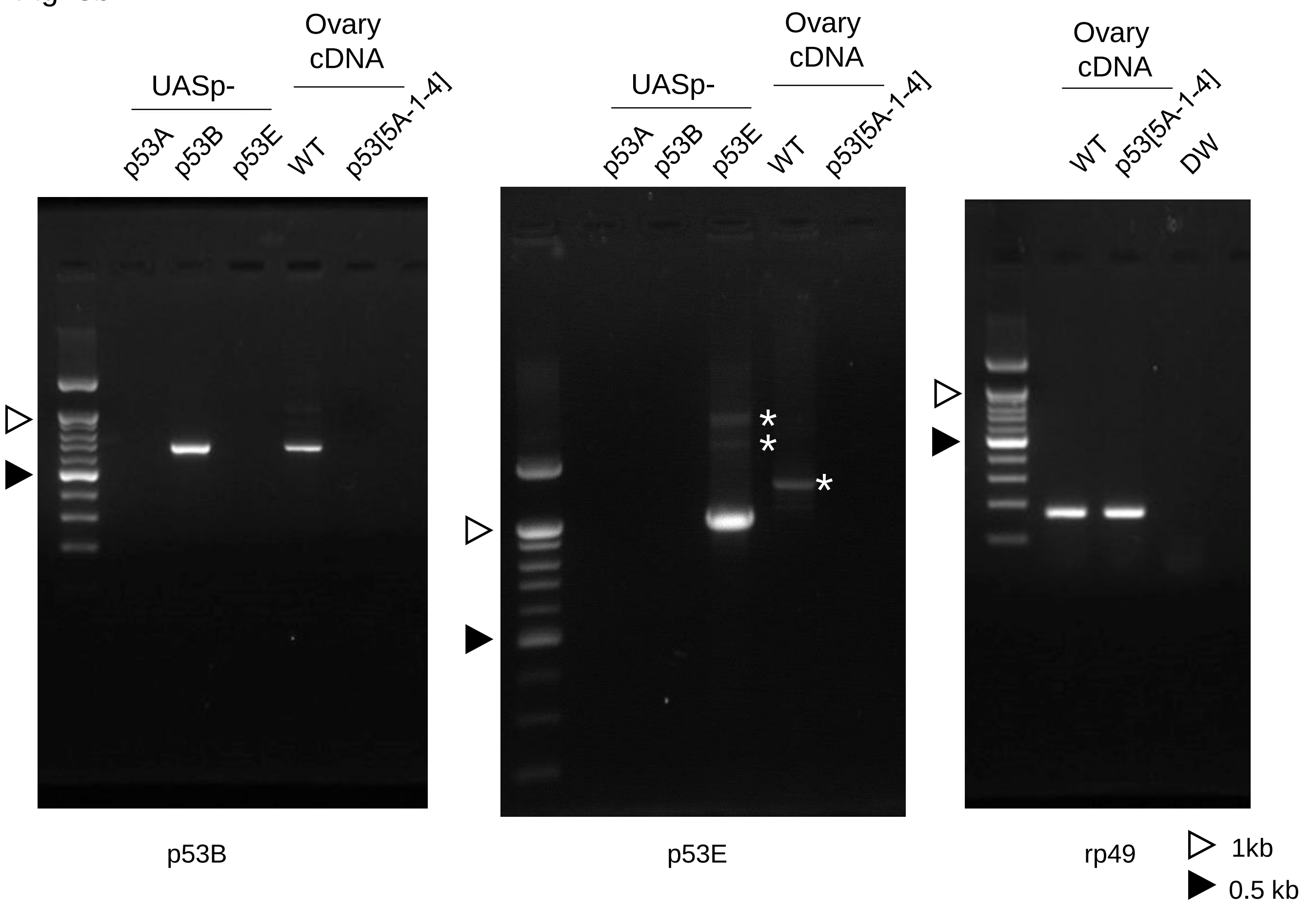

Bands indicated with * may be non-specific signal.

Fig. 3c

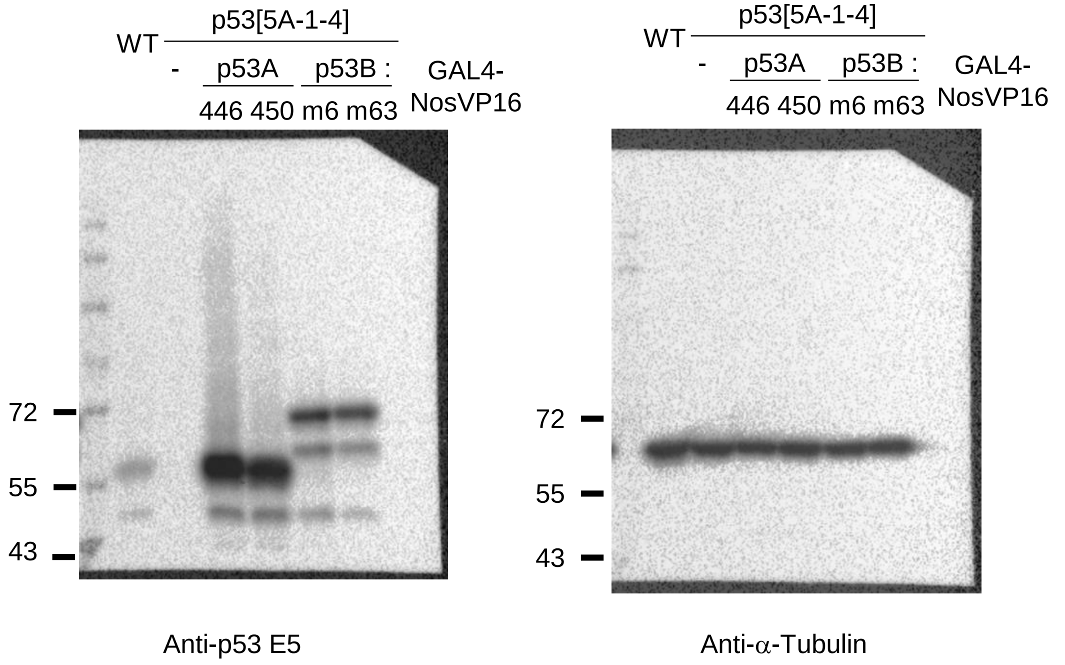

The membrane immunoblotted with anti-p53 antibody was stripped and reprobed with anti-α-Tubulin antibody.

Fig. S5. Uncropped images of western blots of Fig. 4a.

Fig. 4a

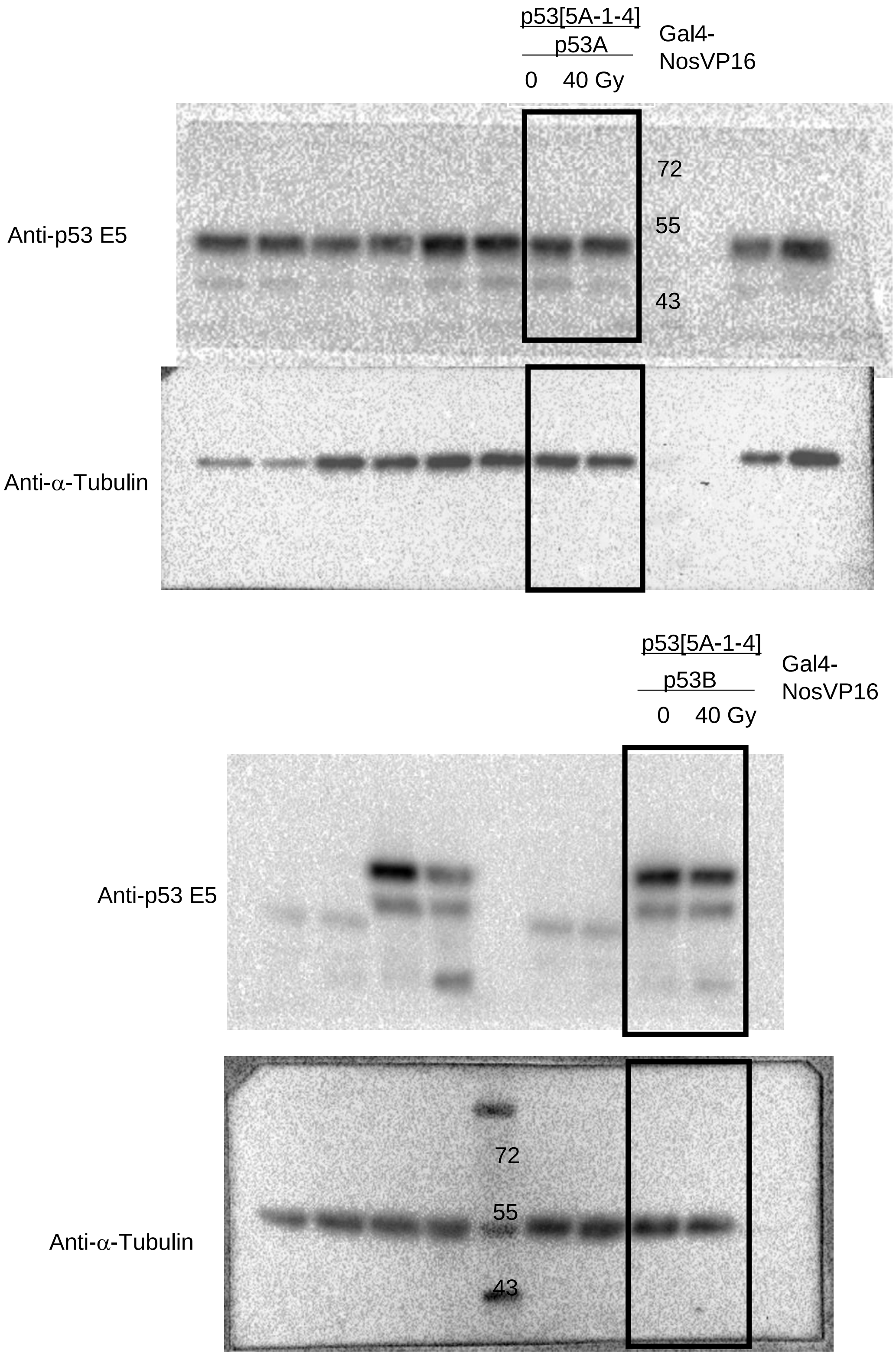

The membrane immunoblotted with anti-p53 antibody was stripped and reprobed with anti-α-Tubulin antibody.

Fig. S5. Uncropped images of western blots of Fig. 4b and 6a.

Fig. 4b

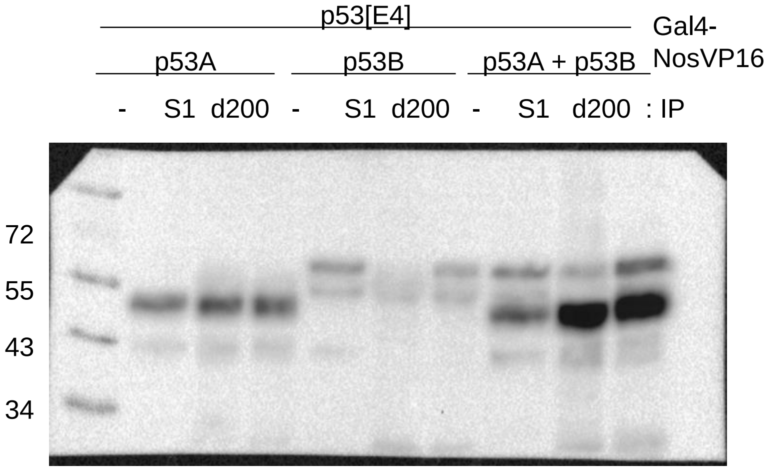

Fig. 6a

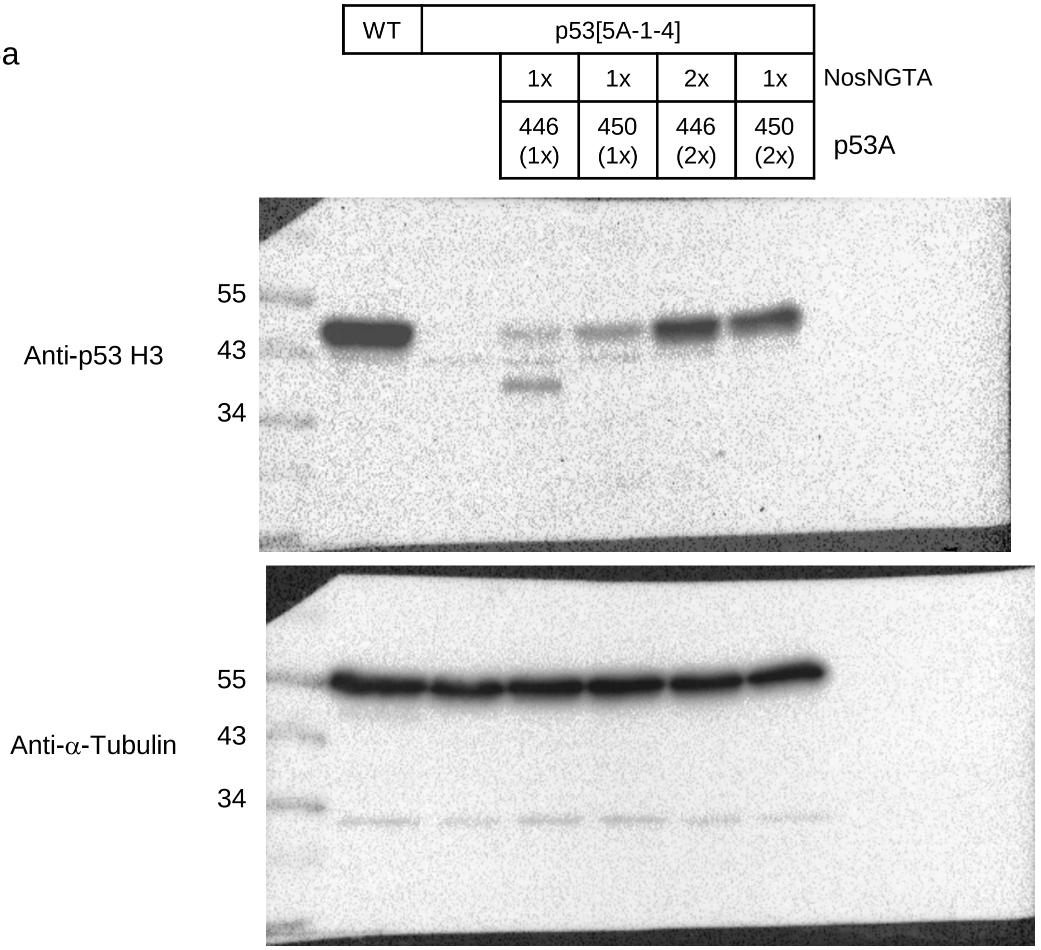

The membrane immunoblotted with anti-p53 antibody was stripped and reprobed with anti-α-Tubulin antibody.
